# Pelvic Organ Prolapse Symptoms in Female Runners: A Cross Sectional Study

**DOI:** 10.1007/s00192-026-06584-w

**Published:** 2026-03-17

**Authors:** Ana Paula Magalhães Resende, Wanessa Silva de Oliveira, Ana Carla Mendes dos Reis, Rafaela de Melo Silva, Maíta Poli Araújo, Marair Gracio Ferreira Sartori

**Affiliations:** 1 Physiotherapy Post-graduation Program, Federal University of Uberlândia, R. Benjamin Constant, 1286, Uberlândia, Minas Gerais Zip Code: 38400-678 Brazil; 2 Gynecology Department, Federal University of São Paulo (UNIFESP-EPM), São Paulo, Brazil

**Keywords:** Pelvic organ prolapse, Running, Pelvic floor disorders, Exercise

## Abstract

**Introduction and Hypothesis:**

Female participation in long-distance running has been increasing, raising concern about pelvic floor-related symptoms in this population. However, data on pelvic organ prolapse (POP) symptoms, particularly during running, remain limited. We hypothesized that higher training volume would be associated with greater prevalence of POP symptoms. This study aimed to determine the prevalence of POP symptoms and associated factors among Brazilian female runners.

**Methods:**

In this cross-sectional study, participants were recruited at road-running events and completed a questionnaire on demographics, health history, training characteristics, and other physical activities. POP symptoms were assessed by two questions: (1) “Do you notice a bulge or lump in your vagina in daily life situations, outside of physical exercise?” and (2) “Do you feel a sensation of pressure, bulge, or lump descending in your vagina while running?”

**Results:**

Three hundred and one women met inclusion criteria (mean age 37.2 years). Twenty-three percent reported POP symptoms during running; 58% had a history of pregnancy, with mean values of 0.77 cesarean and 0.36 vaginal deliveries. Average weekly running distance was 28.6 km, and 76% performed additional physical activity (mean 1.98 sessions/week). POP symptoms were significantly associated with weekly running distance (OR 1.03; 95% CI 1.01–1.05) and additional activities (OR 1.53; 95% CI 1.22–1.91), while cesarean delivery was inversely associated (OR 0.42; 95% CI 0.28–0.62).

**Conclusions:**

POP symptoms were more frequent during running than in daily life. Weekly running distance and engagement in additional physical activity were significant predictors, whereas cesarean delivery appeared protective.

## Introduction

Women’s participation in running has been steadily increasing worldwide. Each year, female involvement in long-distance running events grows at a faster rate than male participation [18]. The health benefits are well established, encompassing improvements in diet, sleep, self-care, energy levels, and stress reduction, along with reduced all-cause mortality and enhanced social well-being [10].

Parallel to this growth, sex-specific health concerns have also emerged, including eating disorders [3], sex-related differences in orthopedic injuries [27], menstrual cycle disturbances, and pelvic floor dysfunctions [1, 13, 19, 22].

Pelvic floor dysfunctions reflect impaired function of pelvic muscles and connective structures, manifesting as urinary incontinence, fecal incontinence, sexual dysfunction, and pelvic organ prolapse (POP), the latter defined as the descent of the anterior or posterior vaginal wall or the uterine/vaginal apex in a caudal direction [17]. In the general female population, pelvic organ prolapse is frequently identified on gynecological examination, with anatomical prevalence reaching up to 50%; however, symptomatic POP is reported by approximately 6–10% of women [5, 20, 25]. This population-level prevalence provides an important baseline for interpreting POP symptoms in physically active women.

Specifically in female runners, the prevalence of certain pelvic floor disorders has been documented. Reported urinary incontinence rates range from 30% to 73% [1, 3, 15, 22], while anal incontinence ranges from 34% to 72% [15, 22]. Sexual dysfunction, however, does not appear more prevalent than in the general population [13].

Findings on POP prevalence are inconsistent. The hallmark symptoms include sensations of vaginal bulging or pressure [6, 26], which form the basis of symptom assessment in international consensus definitions and validated questionnaires. Forner et al. [15] reported a prevalence of 12.7% among 521 runners compared with 858 CrossFit participants. In contrast, Sade et al. [22] reported POP symptoms in 68.8% of high-intensity runners and 64.4% of moderate-intensity runners among 180 participants. Both studies utilized the validated PFDI-20 questionnaire, which, while widely used, does not specifically assess symptoms experienced during sports. This limitation is critical, as the pelvic floor sustains greater impact during running than during daily activities, potentially leading to underestimation of symptom burden.

According to Bø et al. [8], the repetitive ground reaction forces generated during running may negatively influence pelvic floor integrity and thereby contribute to POP development in women. Understanding the prevalence and predictors of POP in this population is essential to inform prevention and early intervention strategies.

Thus, the aim of the present study was to assess the prevalence of POP symptoms both during running and in daily life, and to identify associated risk factors by comparing women who run ≤20 km per week with those who run >20 km per week.

## Materials and Methods

This cross-sectional study was approved by the institutional research ethics committee (approval number 74728123.9.0000.5152). All participants provided written informed consent prior to completing an anonymous questionnaire referring to their most recent month of training.

Data collection was conducted through a structured online questionnaire designed to obtain information on demographic characteristics, gynecological and obstetric history, training variables, and symptoms of pelvic organ prolapse (POP). Recruitment occurred in person at running practices and official races, where the investigator approached runners directly and invited them to complete the questionnaire on a mobile device or tablet, accessible via hyperlink or QR code.

Eligibility criteria included age ≥18 years, a minimum of 6 months of consistent running practice, and at least 10 km of weekly running distance. Exclusion criteria comprised neuromuscular disorders, injuries or health conditions that interrupted running for more than 3 weeks in the previous 6 months, current pregnancy, or being within 6 months postpartum, to minimize the influence of physiological postpartum recovery on pelvic floor outcomes. Pelvic floor muscle function and pelvic organ support are known to continue recovering beyond the early postpartum period, with neuromuscular and morphological changes persisting up to 6–12 months after delivery [16].

Regarding to POP symptoms, the most characteristic and most frequently reported symptom in women with pelvic organ prolapse (POP) is the sensation of a vaginal bulge or “something coming down,” which is considered the central clinical marker of this condition [7, 11, 25]. Therefore, pelvic organ prolapse symptoms were assessed in this study using two direct questions: (1) “Do you notice a bulge or lump in your vagina in daily life situations, outside of physical exercise?” and (2) “Do you feel a sensation of pressure, bulge, or lump descending in your vagina while running?”

Validated questionnaires were not employed because currently available instruments (e.g., PFDI-20, POPDI-6, ICIQ-VS) assess pelvic organ prolapse symptoms in daily life contexts and do not capture symptom occurrence during physical exercise or sport. Importantly, the first question used in this study (“Do you notice a bulge or lump in your vagina in daily life situations, outside of physical exercise?”) was directly derived from the International Urogynecological Association/International Continence Society (IUGA/ICS) definition of the cardinal symptom of pelvic organ prolapse, namely the complaint of a vaginal bulge, lump, or sensation of something coming down. This symptom construct is also the core element assessed in validated questionnaires such as the POPDI-6 and ICIQ-VS.

The second question retained the same symptom anchor but contextualized it during running (“…while running”), allowing assessment of exercise-specific symptoms that are not addressed by existing validated tools. This approach represents a conceptually grounded adaptation of an established symptom definition to a sport-specific context, rather than the creation of a novel or unvalidated construct.

Demographic data included age, weight, and height, with body mass index (BMI) subsequently calculated. Gynecological and obstetric history encompassed number of pregnancies, type and number of deliveries, prior pelvic surgeries, and menopausal status. Training-related data included duration of running practice, weekly training frequency, participation in additional physical activities (type and frequency), mean weekly running distance in the previous month, and whether training was self-directed or guided by a professional, mobile app, or coaching group.

The sample size was estimated using G*Power software (version 3.1), considering a 5% significance level and 95% statistical power, based on parameters derived from a previous study conducted in a similar population.

Statistical analysis was performed with jamovi version 2.6.44. Descriptive statistics summarized continuous variables as mean (standard deviation) and categorical variables as frequency (percentage). The Shapiro–Wilk test assessed data normality, and Levene’s test assessed homogeneity of variances. Between-group comparisons were conducted using the Mann–Whitney *U* test for continuous variables and the chi-square test for categorical variables. Weekly running distance was analyzed both as a continuous variable and as a categorical variable using a 20 km/week cutoff (≤20 vs. >20 km/week). This cutoff was defined a priori, based on previous running literature that used 20 km/week to distinguish lower- from higher-volume recreational runners and to reflect meaningful differences in cumulative weekly running exposure (van Poppel et al. [28]).

For predictive analysis, univariate binary logistic regression was applied to explore associations between POP symptoms (present/absent) and potential predictors, including age, BMI, running experience, weekly training frequency, weekly running distance, long runs per week, engagement in additional physical activities, frequency of these activities, pregnancy history, cesarean delivery, and vaginal delivery. Predictors with *p* < 0.20 in the univariate analysis were included in the multivariable model. Only weekly running distance, cesarean delivery, and frequency of additional physical activities remained significant (*p* ≤ 0.05) in the final model. Statistical significance was set at 5%.

## Results

A total of 388 female runners were approached and invited to participate. Although eligibility criteria were checked prior to questionnaire completion, detailed review of the responses revealed that 24 women did not meet the inclusion criteria and were excluded. Specifically, 11 had been running for less than 6 months, seven reported a weekly running distance below 10 km, and six had experienced an injury that interrupted running for more than 3 weeks in the previous 6 months. The final sample therefore comprised 301 participants.

The demographic and clinical characteristics of the sample are summarized in Table 1. The mean age was 37.2 years (±7.9), mean BMI was 21.7 kg/m^2^ (±2.3), and the mean duration of running practice was 50 months (±48.6). The mean number of pregnancies was 1.0 (±0.9), with mean values of 0.6 (±0.8) cesarean and 0.4 (±0.7) vaginal deliveries. Twenty-three women (8%) were postmenopausal.

**Table 1:** Characteristics of the participants

Variable	Mean (±SD)/*n* (%)
Age (years)	37.25 (±7.96)
BMI (kg/m^2^)	21.75 (±2.27)
Pregnancies (IQR)	1 (0.0 to 2.0)
Cesarean deliveries (IQR)	0 (0.0 to 1.0)
Vaginal deliveries (IQR)	0 (0.0 to 1.0)
Duration of running practice (months)	50.03 (±48.61)
Menopause	
Yes	23 (8%)
No	278 (92%)
Sensation of vaginal bulging/heaviness in daily life	
Yes	27 (9%)
No	274 (91%)
Sensation of vaginal bulging/heaviness during running	
Yes	70 (23%)
No	231 (77%)

Values expressed as mean (±SD), *n* (%) or IQR (interquartile range

**Fig. 1:**
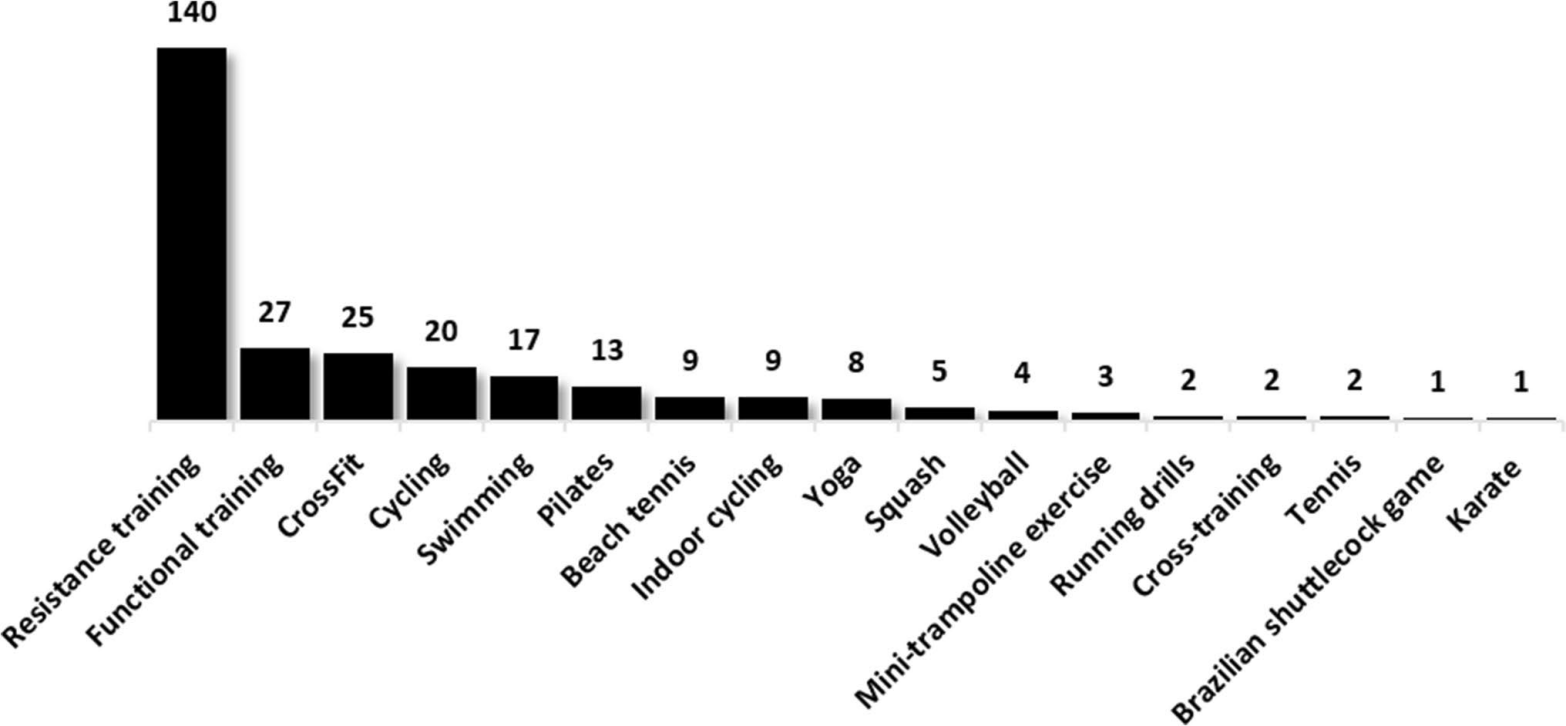
Engagement of female runners in additional exercise beyond running (*n* = 217)

On average, participants reported running 28.6 km per week (±15.6), distributed across 3.3 training sessions (±1.1). While running was the primary activity, 217 women (72%) also engaged in at least one additional form of physical exercise, most frequently strength training, followed by functional training and CrossFit (Fig. 1). The mean frequency of additional physical activity was two sessions per week (±1.4).

Regarding pelvic floor symptoms, 27 women (8%) reported a sensation of vaginal bulging or heaviness in daily life, while 70 women (23%) reported these symptoms specifically during running.

Comparisons between groups according to weekly running distance (≤20 km vs. >20 km) revealed significant differences. Women running more than 20 km per week were older (*p* = 0.004), more often postmenopausal (*p* = 0.036), had longer running experience (*p* < 0.001), trained more frequently (*p* < 0.001), and were more likely to engage in additional physical activity (*p* = 0.004). Conversely, those running ≤20 km per week had higher BMI (*p* = 0.042), more pregnancies (*p* = 0.025), and a higher number of cesarean deliveries (*p* = 0.037) (Table 2).

**Table 2:** Comparison between female runners according to weekly running distance

Variable	≤ 20 km/week (*n* = 128)	> 20 km/week (*n* = 173)	*p* value
Age (years)	35.73 (±6.75)	38.36 (±8.59)	0.004*a
BMI (kg/m^2^)	22.18 (±2.86)	21.43 (±1.64)	0.042*a
Pregnancies (IQR)	**1.0 (0.0 to 2.0)**	**1.0 (0.0 to 2.0)**	0.025*a
Cesarean deliveries (IQR)	**0.0 (0.0 to 1.0)**	**0.0 (0.0 to 1.0)**	0.037*a
Vaginal deliveries (IQR)	**0.0 (0.0 to 0.25)**	**0.0 (0.0 to 1.0)**	0.693a
Menopausal status			0.036*b
Yes	5 (2%)	18 (6%)	
No	123 (41%)	155 (51%)	
Duration of running practice (months)	24.86 (±19.73)	68.65 (±54.89)	<0.001*a
Weekly running frequency	2.66 (±0.55)	3.91 (±1.16)	<0.001*a
Additional physical activity			0.004*b
Yes	105 (35%)	116 (39%)	
No	23 (8%)	57 (19%)	
POP symptoms in daily life			0.313b
Yes	9 (3%)	18 (6%)	
No	119 (40%)	155 (51%)	
POP symptoms during running			0.833b
Yes	29 (10%)	41 (14%)	
No	99 (33%)	132 (44%)	

Values expressed as mean (±SD), *n* (%), or IQR (interquartile range). *BMI* Body mass index. Significant results at *p* < 0.05 are marked with an asterisk.

aMann–Whitney *U* test; b chi-square test

In the multivariable analysis, weekly running distance and frequency of additional physical activity were positively associated with POP symptoms during running, whereas cesarean delivery was inversely associated, reducing the likelihood of symptoms (Table 3). Each additional kilometer per week increased the odds of symptoms by 3% (OR 1.03; 95% CI 1.01–1.05; *p* = 0.001), and each increase in the frequency of additional physical activity raised the odds by 53% (OR 1.53; 95% CI 1.22–1.91; *p* < 0.001). Cesarean delivery reduced the likelihood of symptoms by 58% (OR 0.42; 95% CI 0.28–0.62; *p* < 0.001).

**Table 3:** Adjusted odds ratios for the association between POP symptoms and risk factors

Risk factors	OR (95% CI)	*p* value
Weekly running distance (km/week)	1.03 (1.01–1.05)	0.001*
Frequency of additional physical activity	1.53 (1.22–1.91)	<0.001*
Cesarean delivery	0.42 (0.28–0.62)	<0.001*

Binary logistic regression, Significant results at p<0.05 are marked with an asterisk

## Discussion

This study examined the prevalence of pelvic organ prolapse (POP) symptoms during running and activities of daily living (ADLs). POP symptoms were reported by 23% of participants during running but only by 8% during ADLs. Weekly running distance and the frequency of additional physical activity were identified as significant predictors, whereas cesarean delivery appeared to be a protective factor.

Regarding prevalence, prior studies have reported highly variable rates of POP among female athletes. Forner et al. [15] documented a prevalence of 12.7% in runners, whereas Sade et al. [22] reported much higher figures—64.4% and 68.8% in moderate- and high-intensity runners, respectively. In the present study, prevalence was intermediate at 23%. In population-based studies, symptomatic pelvic organ prolapse affects approximately 6–10% of women [5, 20, 25]. When contrasted with this baseline, the prevalence observed during running in our cohort suggests a higher symptom burden under conditions of physical stress. Although all studies focused on amateur but experienced runners, discrepancies may reflect the absence of standardized instruments to assess pelvic floor symptoms in athletes. Both Forner et al. [14] and Sade et al. utilized the validated Pelvic Floor Distress Inventory (PFDI-20), which comprehensively assesses pelvic floor dysfunctions but does not specifically capture symptom occurrence during sports. As a result, symptom prevalence may be underestimated in athletic cohorts [4].

Our findings further highlight that symptom perception may vary by context. When asked specifically about symptoms during running and during ADLs, participants reported substantially different prevalence rates (23% vs. 8%). This difference likely reflects the greater biomechanical demands imposed on the pelvic floor during running compared to daily tasks. Early-stage prolapse is often asymptomatic, as organ descent remains within the less innervated proximal vaginal canal [5, 20]. The higher prevalence during running observed in our study may therefore be explained by pelvic floor fatigue and reduced neuromuscular responsiveness during impact activities, impairing organ support and provoking sensations of pressure or bulging [21].

In terms of predictors, Campbell et al. [9] found no significant association between training variables and POP among 1141 recreational athletes and 457 non-athletes. In contrast, our findings identified weekly running distance and frequency of additional physical activity as significant risk factors. This divergence may relate to the heterogeneity of sports and training volumes in Campbell et al.’s cohort, or to the use of the ICIQ-VS questionnaire, which, while validated, does not assess sport-specific symptoms.

Although our study did not use a standardized questionnaire, it directly addressed the cardinal POP symptom, vaginal bulging, defined as a sensation of pressure or a lump, and specifically inquired about its occurrence during running. This symptom has been shown to correlate most strongly with clinical diagnosis of POP [6, 26]. In fact, the most characteristic and most frequently reported symptom in women with pelvic organ prolapse (POP) is the sensation of a vaginal bulge or “something coming down,” which is considered the central clinical marker of this condition [7, 11, 25].

When it comes to runners, it is very common to engage in additional forms of exercise, and our findings were no exception. Our data showed that participation in other exercise modalities was associated with a higher frequency of POP symptoms. Among the participants, the most frequently reported activity besides running was strength/resistance training. Similarly, Loudon et al. (2022) found that 77.9% of female master runners performed strength training, followed by cycling (64.7%) and swimming (47.1%)[18]. Consistent with our results, Santos et al. [23] reported that among 801 Brazilian recreational runners, 85.4% of women incorporated strength training into their routines. This reflects the well-established benefits of strength training for runners, including injury prevention and improved running economy [23]. On the other hand, accumulating high weekly training volumes has been associated with a greater prevalence of urinary incontinence [2].

Cesarean delivery emerged as a protective factor in our analysis. This finding is consistent with a systematic review and meta-analysis of 47,429 women that confirmed cesarean birth as protective against POP in the general population [24]. However, in our study, vaginal delivery was not identified as a direct risk factor. While pelvic floor neuromuscular trauma from vaginal delivery has been implicated in POP pathogenesis, these findings do not warrant cesarean delivery as the preferred mode of birth.

To our knowledge, no prior studies have demonstrated positive associations between weekly running distance or additional exercise frequency and POP, as found here. However, training volume has been linked to stress urinary incontinence, suggesting that the intensity and cumulative load of exercise may similarly influence POP risk, warranting further investigation.

Despite the observed associations, exercise remains fundamental for women’s health. The identification of POP symptoms should not discourage participation but rather guide preventive and educational strategies. As women’s participation in running continues to expand, pelvic health awareness and early management are critical to sustaining engagement in sport. Previous research has shown that women with pelvic floor dysfunctions may withdraw from physical activity due to embarrassment [12], underscoring the importance of proactive interventions.

Strengths of the present study include its focus on POP symptoms specifically in runners, the distinction between symptoms during sport and daily life, and in-person recruitment at official running events, ensuring a well-characterized sample of experienced amateur athletes. Limitations include the absence of gynecological examination or objective pelvic floor assessment, and the relatively high prevalence of cesarean births, which reflects the elevated national cesarean rate in Brazil and may limit generalizability.

## Conclusion

In this cohort of Brazilian female runners, the prevalence of POP symptoms was 23% during running and 8% during activities of daily living. Weekly running distance and frequency of additional physical activity were identified as significant predictors of prolapse symptoms, whereas cesarean delivery was inversely associated and appeared protective. These findings highlight the need for tailored preventive strategies and pelvic health education to support women’s safe and sustained participation in endurance running.

## Data Availability

The datasets generated and analyzed during the current study are available from the corresponding author on reasonable request.

## References

[1] Abitteboul Y, Leonard F, Mouly L, Riviere D, Oustric S. Incontinence urinaire chez des coureuses de loisir de marathon. Prog Urol. 2015;25(11):636–41. 26159054 10.1016/j.purol.2015.05.009

[2] Alves JO, Luz STD, Brandão S, da Luz CM, Jorge RN, da Roza T. Urinary incontinence in physically active young women: prevalence and related factors. Int J Sports Med. 2017;38(12):937–41. 28950397 10.1055/s-0043-115736

[3] Araújo MP, Oliveira ED, Zucchi EV, Trevisani VF, Girão MJ, Sartori MG. The relationship between urinary incontinence and eating disorders in female long-distance runners. Rev Assoc Med Bras. 2008;54(2):146–9. 18506324 10.1590/s0104-42302008000200018

[4] Arouca MA, Duarte TB, Lott DA, Magnani PS, Nogueira AA, Rosa-e-Silva JC, et al. Validation and cultural translation for Brazilian Portuguese version of the Pelvic Floor Impact Questionnaire (PFIQ-7) and Pelvic Floor Distress Inventory (PFDI-20). Int Urogynecol J. 2016;27(7):1097–106. 26782099 10.1007/s00192-015-2938-8

[5] Barber MD, Maher C. Epidemiology and outcome assessment of pelvic organ prolapse. Int Urogynecol J. 2013;24(11):1783–90. 24142054 10.1007/s00192-013-2169-9

[6] Barber MD, Neubauer NL, Klein-Olarte V. Can we screen for pelvic organ prolapse without a physical examination in epidemiologic studies? Am J Obstet Gynecol. 2006;195(4):942–8. 16681989 10.1016/j.ajog.2006.02.050

[7] Barber MD, Maher C, Bø K. An International Urogynecological Association (IUGA)/International Continence Society (ICS) joint report on the terminology for female pelvic organ prolapse (POP). Neurourol Urodyn. 2016;35(2):137–68. 26749391 10.1002/nau.22922

[8] Bø K, Anglès-Acedo S, Batra A, Brækken IH, Chan YL, Jorge CH, et al. Strenuous physical activity, exercise, and pelvic organ prolapse: a narrative scoping review. Int Urogynecol J. 2023;34(6):1153–64. 36692525 10.1007/s00192-023-05450-3 PMC10238337

[9] Campbell KG, Batt ME, Drummond A. Prevalence of pelvic floor dysfunction in recreational athletes: a cross-sectional survey. Int Urogynecol J. 2023 Oct;34(10):2429–2437. 10.1007/s00192-023-05548-8 10.1007/s00192-023-05548-8 PMC10590299 37162534

[10] Chakravarty EF, Hubert HB, Lingala VB, Fries JF. Reduced disability and mortality among aging runners: a 21-year longitudinal study. Arch Intern Med. 2008;168(15):1638–46. 18695077 10.1001/archinte.168.15.1638 PMC3175643

[11] Cooper J, Annappa M, Drutz HP, Lamping DL, Black NA. Prevalence of genital prolapse symptoms in primary care: a cross-sectional survey. Int Urogynecol J. 2015;26(4):505–10. 25381004 10.1007/s00192-014-2556-x

[12] Dakic JG, Cook J, Hay-Smith J, Lin KY, Frawley H. Pelvic floor disorders stop women exercising: a survey of 4556 symptomatic women. J Sci Med Sport. 2021;24(12):1211–7. 34244084 10.1016/j.jsams.2021.06.003

[13] de Melo SR, da Roza TH, Secchi LLB, da Silva Serrão PRM, Resende APM. Can running influence women’s sexual function? Int Urogynecol J. 2023;34(4):905–11. 35798997 10.1007/s00192-022-05266-7

[14] Forner LB, Beckman EM, Smith MD. Symptoms of pelvic organ prolapse in women who lift heavy weights for exercise: a cross-sectional survey. Int Urogynecol J. 2020;31(8):1551–8. 31813038 10.1007/s00192-019-04163-w

[15] Forner LB, Beckman EM, Smith MD. Do women runners report more pelvic floor symptoms than women in CrossFit®? A cross-sectional survey. Int Urogynecol J. 2021;32(2):295–302. 32955598 10.1007/s00192-020-04531-x

[16] Goom T, Donnelly G, Brockwell E. Returning to running postnatal—guidelines for medical, health and fitness professionals. J Womens Health Phys Ther. 2019;43(2):57–64.

[17] Haylen BT, Maher CF, Barber MD, Camargo S, Dandolu V, Digesu A, et al. An International Urogynecological Association (IUGA)/International Continence Society (ICS) joint report on the terminology for female pelvic organ prolapse (POP). Int Urogynecol J. 2016;27(2):165–94. 26755051 10.1007/s00192-015-2932-1

[18] Loudon J, Parkerson-Mitchell A. Training habits and injury rate in masters female runners. Int J Sports Phys Ther. 2022;17(3):501–7. 35391857 10.26603/001c.32374 PMC8975576

[19] Lynch SL, Hoch AZ. The female runner: gender specifics. Clin Sports Med. 2010;29(3):477–98. 20610034 10.1016/j.csm.2010.03.003

[20] Nygaard I, Barber MD, Burgio KL, Kenton K, Meikle S, Schaffer J, et al. Prevalence of symptomatic pelvic floor disorders in US women. JAMA. 2008;300(11):1311–6. 18799443 10.1001/jama.300.11.1311 PMC2918416

[21] Ree ML, Nygaard I, Bø K. Muscular fatigue in the pelvic floor muscles after strenuous physical activity. Acta Obstet Gynecol Scand. 2007;86(7):870–6. 17611834 10.1080/00016340701417281

[22] Sade S, Naor I, Rotem R, Waichenberg L, Kravits DZ, Weintraub AY. Pelvic floor disorders among amateur runners. Arch Gynecol Obstet. 2024;309(5):2223–8. 38341841 10.1007/s00404-023-07351-8

[23] Santos WKA, Forte LDM, Silva AS, Rufino HVO, Vieira LF, Silva JMFL, et al. Habits related to strength training of Brazilian recreational runners. Sports. 2024;13(1):3. 10.3390/sports13010003. 39852599 10.3390/sports13010003 PMC11768657

[24] Schulten SFM, Claas-Quax MJ, Weemhoff M, van Eijndhoven HW, van Leijsen SA, Vergeldt TF, et al. Risk factors for primary pelvic organ prolapse and prolapse recurrence: an updated systematic review and meta-analysis. Am J Obstet Gynecol. 2022;227(2):192–208. 35500611 10.1016/j.ajog.2022.04.046

[25] ten Slieker-Hove MCP, Pool-Goudzwaard AL, Eijkemans MJC, Steegers-Theunissen RPM, Burger CW, Vierhout ME. The prevalence of pelvic organ prolapse symptoms and signs and their relation with bladder and bowel disorders in a general female population. Int Urogynecol J. 2009;20(9):1037–45. 10.1007/s00192-009-0902-1. 10.1007/s00192-009-0902-1 PMC2721135 19444368

[26] Tan JS, Lukacz ES, Menefee SA, Powell CR, Nager CW. Predictive value of prolapse symptoms: a large database study. Int Urogynecol J. 2005;16(3):203–9. 10.1007/s00192-004-1243-8 15875236

[27] van der Worp MP, ten Haaf DS, van Cingel R, de Wijer A, van der Nijhuis- Sanden MW, Staal JB. Injuries in runners: a systematic review on risk factors and sex differences. PLoS One. 2015;10(2):e0114937. 25706955 10.1371/journal.pone.0114937 PMC4338213

[28] van Poppel D, van der Worp M, Slabbekoorn A, van den Heuvel SSP, van Middelkoop M, Koes BW, Verhagen AP, Scholten-Peeters GGM. Risk factors for overuse injuries in short- and long-distance running: A systematic review. J Sport Health Sci. 2021 Jan;10(1):14–28. 10.1016/j.jshs.2020.06.006 10.1016/j.jshs.2020.06.006 PMC7856562 32535271

